# Thyroid surgery during the COVID-19 pandemic: results from a systematic review

**DOI:** 10.1007/s40618-021-01641-1

**Published:** 2021-07-19

**Authors:** L. Scappaticcio, M. I. Maiorino, S. Iorio, C. Camponovo, A. Piccardo, G. Bellastella, G. Docimo, K. Esposito, P. Trimboli

**Affiliations:** 1 Division of Endocrinology and Metabolic Diseases, University of Campania “L. Vanvitelli”, 80138 Naples, Italy; 2 Department of Medical and Advanced Surgical Sciences, University of Campania “L. Vanvitelli”, Naples, Italy; 3 Clinic for Endocrinology and Diabetology, Lugano Regional Hospital, Ente Ospedaliero Cantonale, Lugano, Switzerland; 4 Department of Nuclear Medicine, E.O. Ospedali Galliera, Genoa, Italy; 5 Division of Thyroid Surgery, University of Campania “L. Vanvitelli”, Naples, Italy; 6 Faculty of Biomedical Sciences, Università della Svizzera Italiana (USI), Lugano, Switzerland

**Keywords:** Thyroid, Surgery, COVID-19

## Abstract

**Purpose:**

During the COVID-19 pandemic, elective thyroid surgery is experiencing delays. The problem is that the COVID-19 pandemic is ongoing. The research purposes were to systematically collect the literature data on the characteristics of those thyroid operations performed and to assess the safety/risks associated with thyroid surgery during the COVID-19 pandemic.

**Methods:**

We used all the procedures consistent with the PRISMA guidelines. A comprehensive literature in MEDLINE (PubMed) and Scopus was made using ‘‘Thyroid’’ and “coronavirus” as search terms.

**Results:**

Of a total of 293 articles identified, 9 studies met the inclusion criteria. The total number of patients undergoing thyroid surgery was 2217. The indication for surgery was malignancy in 1347 cases (60.8%). Screening protocols varied depending on hospital protocol and maximum levels of personal protection equipment were adopted. The hospital length of stay was 2–3 days. Total thyroidectomy was chosen for 1557 patients (1557/1868, 83.4%), of which 596 procedures (596/1558, 38.3%) were combined with lymph node dissections. Cross-infections were registered in 14 cases (14/721, 1.9%), of which three (3/721, 0.4%) with severe pulmonary complications of COVID-19. 377 patients (377/1868, 20.2%) had complications after surgery, of which 285 (285/377, 75.6%) hypoparathyroidism and 71 (71/377, 18.8%) recurrent laryngeal nerve injury.

**Conclusion:**

The risk of SARS-CoV-2 transmission after thyroid surgery is relatively low. Our study could promote the restart of planned thyroid surgery due to COVID-19. Future studies are warranted to obtain more solid data about the risk of complications after thyroid surgery during the COVID-19 era.

**Supplementary Information:**

The online version contains supplementary material available at 10.1007/s40618-021-01641-1.

## Introduction

The COVID-19 pandemic continues to affect all aspects of healthcare in Europe and abroad.

Elective surgeries have been almost totally postponed to keep to a minimum the risk of transmission of SARS-CoV-2 and also to allow a better allocation of resources [1, 2]. Since thyroid surgery usually does not cover immediate surgical interventions, during the COVID-19 pandemic nearly all of the patients who require thyroid surgery care are experiencing delays in the operation planning procedure [3–6]. This scenario has the potential to cause negative outcomes and anxiety in patients with a diagnosis of thyroid cancer, nodules with indeterminate cytology, already scheduled surgery for hyperthyroidism. Likewise, it is of higher value in this times to remind patients about the generally good/excellent prognosis associated with differentiated thyroid cancer, the role that active surveillance can play in low-risk papillary thyroid cancer (PTC) and the possibility to alleviate symptoms of hyperthyroidism through medical therapy [3, 7]. Despite all this, the diffusion and the acceptability of less invasive management options, including active surveillance of PTC, seems to still be quite limited and variable depending on country, medical, and health culture environment [3]. Now, the main problem is that the COVID-19 pandemic is ongoing and the end is not in sight. This context could determine further postponement or cancellation of elective thyroid surgery resulting in potential negative effects on patient management.

International experts have issued statements and guidelines addressing the optimal approach to performing thyroid surgery during this time [8–14]. Briefly, urgent surgery should be considered for the following reasons: thyroid cancers that exhibit aggressive tumor biology or local invasion; severely symptomatic Graves’ hyperthyroidism that cannot be medically controlled; goiters with symptomatic airway compromise [8–13]. Non-urgent interventions, on the other hand, are deemed to be those regarding: small differentiated thyroid cancers; indeterminate thyroid nodules; hyperthyroidism, and goiters not associated to immediate health concerns [8–14]. All these recommendations represent opinions and general principles from experts in the field, and they are not made on a case-by-case basis considering individual patient factors and regional and local hospital resource capacity.

The present systematic review was conceived to explore the worldwide experience of Surgery Divisions in managing thyroid diseases during the COVID-19 pandemic. Particularly, the research purposes were to systematically collect together the literature data on the characteristics of those thyroid operations performed and to assess the safety/risks associated with thyroid surgery during the COVID-19 pandemic.

## Methods

### Conduction of the systematic review

In this study, we used all the procedures consistent with the Preferred Reporting Items for Systematic Reviews and Meta-Analyses (PRISMA) guidelines [15]. Human subjects or the public were not involved in any way in our study.

### Search strategy

Two investigators (L.S. and P.T.) independently conducted a comprehensive literature search in the online databases of MEDLINE (PubMed) and Scopus using the following search terms and their combinations: ‘‘Thyroid’’ and “coronavirus” (or “SARS-CoV-2” or “COVID-19”). A commencement date limit was not used, and the last search was carried on April 25, 2021. No language restrictions were imposed. The search strategy was refined to evaluate all references of the screened literature to identify additional relevant studies. The search was restricted to human studies.

### Eligibility criteria and study selection

Records identified by our search strategy were screened using ‘‘the report of thyroid surgery during the COVID-19 pandemic’’ as the major criterion of inclusion. Eligible cohorts should correspond to patients undergoing thyroid surgery during the COVID-19 pandemic. Only research articles were considered for inclusion (i.e., experimental studies, observational studies, and case series). Excluded studies were: case reports, reviews or guidelines, editorials, letters, commentaries, and meeting abstracts.

Two researchers (L.S. and P.T.), applying the above criteria, independently reviewed titles and abstracts of the screened articles. Then, all authors independently reviewed the main text of the eligible articles to define their inclusion. Disagreements were resolved by consensus among all the reviewers.

### Data extraction

For the included studies, the following data were coded and extracted independently and in duplicate by two investigators (L.S. and P.T.), in a piloted form: (I) author, country, study design, community SARS-CoV-2 data; (II) number of patients undergoing thyroid surgery, of which sex and age were reported; (III) reason of thyroid surgery (i.e. benign versus malignant thyroid diseases); (IV) use of screening protocols and protective equipment for COVID-19; (V) average hospital stay and length of thyroid surgery; (VI) type of surgical procedure; (VII) follow-up period after surgery; (VIII) number of cross-infections (i.e. from patient to medical staff and vice versa) with the SARS-CoV-2 virus and their severity; (IX) number and types of complications of thyroid surgery.

### Risk of bias assessment

The risk of bias of the included studies was assessed independently by two investigators (L.S. and P.T.) through the National Heart, Lung, and Blood Institute Quality Assessment Tool (https://www.nhlbi.nih.gov/health-topics/study-quality-assessment-tools).

### Study endpoints

The primary outcome was to explore the main features of surgery setting where thyroid interventions were performed: i.e. community SARS-CoV-2 scenario, indication for thyroid surgery (benign or malignant thyroid disease), screening protocols for SARS-CoV-2 detection, personal protection equipment used, hospital length of stay, duration, and type of surgical procedures. The secondary outcome was to assess the safety/risks associated with thyroid surgery during the COVID-19 pandemic, using as estimates cross-infections with SARS-CoV-2 and complications after surgery.

## Results

### Study selection

**Fig. 1:**
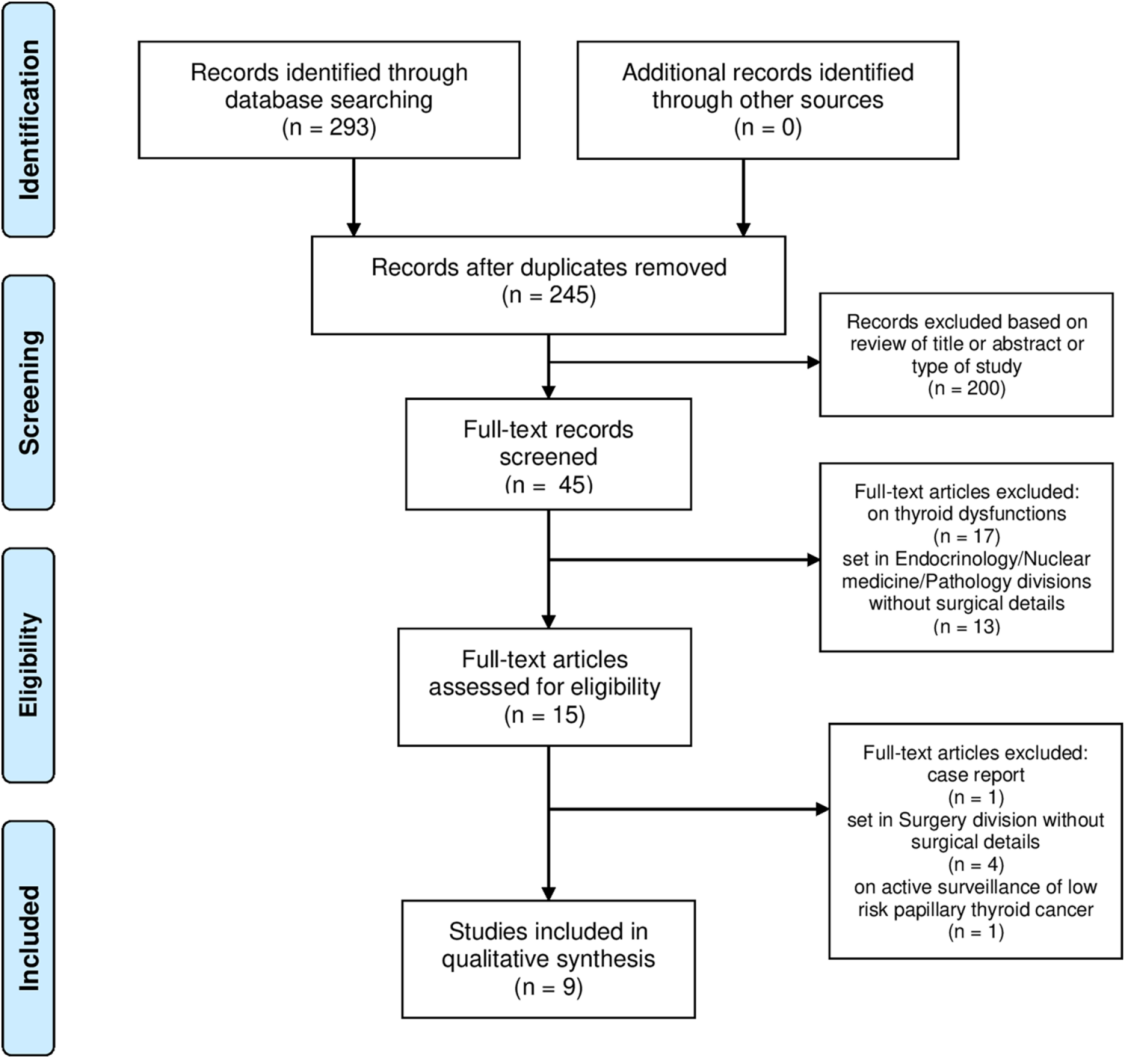
Flowchart of study selection process

The literature search using the above algorithm yielded 293 studies. All the studies assessed and the reasons for exclusion are shown in Fig. 1. Among the studies finally excluded, there were four studies without details on thyroid surgery (one set in Vietnam [16], one in Italy [17], one International [18], one in Italy-UK [19]), one relative to an operated case of PTC [20], one regarding potential surgical candidates who underwent active surveillance [21]. Nine English language studies had appropriate data for systematic review [22–30].

### Systematic review

Table 1 details the features of the nine included studies. These studies were performed across several countries: three in China [23, 27, 28], three in Italy [25, 29, 30], one in USA [26], one in Jordan [22], while the remaining one enrolled a cohort of patients from 26 countries [24]. They were seven single-center observational cohort studies [22, 23, 25–29] and two were multi-center studies [24, 30], all with a retrospective study design except for two prospective studies [24, 28]. Overall, the studies took place between January and August 2020 in countries with community SARS-CoV-2 prevalence varying from low to high. The total number of patients undergoing thyroid surgery was 2217, they were aged from 11 to 80 years, 1531 were females and 571 males (female/male ratio ~ 3, with gender reported in five studies [22, 24, 27, 28, 30]). The indication for surgery was malignancy in 1347 (60.8%) and benign thyroid disease in 870 (39.2%) cases. The size of the primary lesion could vary from occult [22] to more than 40 mm [22, 24, 27]. Screening protocols for SARS-CoV-2 detection in hospitalized patients could be based only on COVID-19-related symptoms [22, 26] or on also swab and blood tests [25, 30] and chest computed tomography (CT) [23, 24, 27–29]. Personal protection equipment consisting of N95 masks, eye protection, gloves, and gowns was adopted by medical staff in all the studies. The average hospital length of stay was three days in four studies [25–27, 30] and two days in one study [28]. The mean operating time was reported by two studies [27, 30], and it varied from 57 to 90 min. Follow-up time after surgery could vary from two [23, 29] to four weeks [24, 26]. Details regarding the adopted surgical procedures were reported by five studies [22, 25, 27, 28, 30]: total thyroidectomy was chosen for 1557 patients (1557/1868, 83.4%), of which 596 procedures (596/1557, 38.3%) were combined with central and/or lateral lymph node dissections. Hemithyroidectomy was the elective surgical procedure for 311 patients (311/1868, 16.6%). Among 721 patients of seven studies [22–24, 26–29] cross-infections were registered in 14 cases (14/721, 1.9%), of which three (3/721, 0.4%) with severe pulmonary complications of COVID-19 [24]. Four studies [22, 27, 28, 30] reported data on complications: they showed that 377 patients (377/1868, 20.2%) had complications after surgery, of which 285 (285/377, 75.6%) had hypoparathyroidism and 71 (71/377, 18.8%) had recurrent laryngeal nerve injury.

**Table 1:** Main features of the 9 included studies

Authors	Bakkar et al. [22]	Cai et al. [23]	COVIDSurg Coll. [24]	Lombardi et al. [25]	Wai et al. [26]	Zhang et al. [27]	Zhao et al. [28]	Ferrari et al. [29]	Medas et al. [30]	Total, *n* (%)
Country	Jordan	China	26 countries	Italy	USA	China	China	Italy	Italy	
Study design	R	R	P	R	R	R	P	R	R	
Timeframe (mth/y)	3–5/2020	2–3/2020	6–8/2020	3/2020	3–4/2020	1–4/2020	3–6/2020	3–4/2020	3–8/2020	
Community SARS-CoV-2 prevalence	Low	High	Low/high	High	High	High	High	High	High	
Undergoing Surgery (*n*)	12	86	235	18	2	328	50	8	1478	2217
Age (y)	56 (33–80)	n.a.	20–79	n.a.	n.a.	43 ± 11	11–39	n.a.	51	
Female/Male (*n*)	9/3	n.a.	151/83	n.a.	n.a.	271/57	40/10	n.a.	1060/418	1531/571
Malignant (M) or Benign (B) (*n*)	12 (M)	86 (M)	235 (M)	14 (M), 4 (B)	2 (M)	293 (M), 35 (B)	37 (M), 13 (B)	8 (M)	662 (M), 816 (B)	1347 (60.8) (M), 870 (39.2) (B)
Tumor size (mm)	44 (0–80)	n.a.	< 20, > 40	n.a.	n.a.	3.9 ± 1.4	n.a.	n.a.	18	
Screening protocols	Symptoms	Symptoms, blood tests, chest CT	Symptoms, blood tests, swab tests, chest CT	Symptoms, swab test	Symptoms	Symptoms, swab tests, chest CT	Symptoms, blood tests, swab tests, chest CT	Symptoms, blood tests, swab tests, chest CT	Swab tests	
Protective equipment	maxPPE	maxPPE	maxPPE	n.a.	maxPPE	maxPPE	maxPPE	maxPPE	maxPPE	
Average hospital stay (days)	n.a.	n.a.	n.a.	3	3	3 ± 1	2	n.a.	3	
Mean operating time (min)	n.a.	n.a.	n.a.	n.a.	n.a.	57 ± 12	n.a.	n.a.	90	
Thyroidectomy (TT) or hemithyroidectomy (ET) (*n*)	11 (TT), 1 (ET)	n.a.	n.a.	18 (TT), 0 (ET)	n.a.	328 (TT), 0 (ET)	32 (TT), 18 (ET)	n.a.	1186 (TT), 292 (ET)	1557 (83.4) (TT), 311 (16.6) (ET)
Lymph node dissections (*n*)	12	n.a.	n.a.	n.a.	n.a.	321	6	n.a.	257	596 (38.3)
F.U. period after surgery (weeks)	n.a.	2	4	n.a.	4	n.a.	n.a.	2	n.a.	
Cross-infections (*n*)	0	0	14	n.a.	0	0	0	0	n.a.	14 (1.9)
Severe COVID-19 (*n*)	0	0	3	n.a.	0	0	0	0	n.a.	3 (0.4)
Complications (*n*)	0	n.a.	n.a.	n.a.	n.a.	35	0	n.a.	342	377 (20.2)
Postoperative Hypoparathyroidism (*n*)	0	n.a.	n.a.	n.a.	n.a.	19	0	n.a.	266	285 (75.6)
Recurrent laryngeal nerve injury (*n*)	0	n.a.	n.a.	n.a.	n.a.	16	0	n.a.	55	71 (18.8)

Age is expressed in years (yr) as mean ± standard deviation (SD) and/or wide range. Tumor size is expressed in mm as mean ± standard deviation (SD) and/or wide range and indicates the maximum diameter. maxPPE includes: N95 masks, eye protection, gloves, and gowns. Hospital stay and operating time are expressed in days and minutes, respectively, as mean ± SD. Complications include hypoparathyroidism, haematoma, recurrent laryngeal nerve injury

*n* number, *mth* month, *y* year, *mm* millimeter, *min* minutes, *F.U.* follow-up, *R* retrospective, *P* prospective, *n.a.* not available, *maxPPE* maximum personal protection equipment, *CT* computed tomography

### Study quality assessment

Supplemental Table S1 summarizes the quality assessment of the nine included studies. The risk of bias for each study could be judged as low in 11 of 14 items. By contrast, all studies did not report anything about power or sample size justification. A participation rate of eligible persons was not mentioned in any of the included studies. In four studies [22, 27, 28, 30], where the follow-up period after surgery was not reported, the risk of bias on outcome measures was regarded as high.

## Discussion

During the Covid-19 pandemic, two main reasons for the postponement of elective thyroid surgery are the risk of transmission of SARS-CoV-2 and the need for a better allocation of health resources [1, 2]. Nevertheless, the current trend of COVID-19 indicates that in large parts of the world this pandemic may continue much longer than expected and may become endemic [31].

The lack of a date from which elective surgery may formally restart and its indefinite postponement could induce negative outcomes and distress of patients with thyroid malignancies or other relevant thyroid disorders [31]. Moreover, this could also cause future thyroid surgery overcrowding and physician burnout [31].

Overall, thyroid surgery is quicker and requires fewer resources than other most demanding surgeries of the head and neck [1, 2, 31]. Current recommendations may not be valid in the scenario where COVID-19 becomes endemic, so we need evidence-based data to support the gradual resumption of elective surgery while living with the SARS-CoV-2 virus [31].

Our paper is the first study aimed at systematically review the experience of thyroid surgeries facing the risk of transmission of SARS-CoV-2 infection. While nine studies [22–30] were finally included for revision, only one study [21] addressed the possibility to safely continue prospective observational research on active surveillance of low-risk papillary thyroid cancer during the COVID-19 pandemic.

Our results are based on a large number of patients undergoing thyroid surgery representative of several countries and different hospital contexts. Thyroid cancer was the indication for surgery in more than 60% of patients, while the rest of the patients were operated on for benign pathology. The size of the primary lesion was very variable, including both microcarcinoma and cancer with local and distant metastases [22, 24, 27]. Total thyroidectomy with combined lymph node dissection was the surgical procedure adopted in about 4 out of 10 patients: noteworthy, this result derived from four studies [22, 27, 28, 30], of which the two studies [27, 28] were set in China where lymph node dissection is a routine practice [32] and the community SARS-CoV-2 prevalence was high from January to June 2020.

The average hospital length of stay was that of conventional thyroid surgery (i.e. 2–3 days) as reported in five studies [25–28, 30]. Also, the time in the operating room was similar to that of thyroid surgery apart to the COVID-19 pandemic, as reported by two studies [27, 30]; however, mean longer periods may be expected since the recommendations to keep the spread of SARS-CoV-2 low in operating rooms [1, 19]. Complications after surgery, including hypoparathyroidism, haematoma, and recurrent laryngeal nerve injury, were found to be higher compared to that usually associated with thyroid surgery before the COVID-19 pandemic [33]: however, most of the complicated cases were due to postoperative hypoparathyroidism, for which it was not known to be transient or permanent. Other possible explanations of the high rate of complications could be the high number of lymph node dissections (i.e., 38.3% of total thyroidectomies, 596/1557) and the greater attention of the surgical team to prevent the spread of infection.

Screening protocols for SARS-CoV-2 detection in hospitalized patients consisted of symptomatic triage in two studies [22, 26] where local community COVID-19 prevalence was very low [22] and COVID-19 testing was not routinely performed during the study period [26], respectively. In the remaining included studies [23–25, 27–30] screening protocols were at least based on swab or blood tests, eventually combined with chest CT. Theoretical maximum levels of personal protection equipment consisting of N95 masks, eye protection, gloves, and gowns were adopted in all the studies. Lastly, we found that SARS-CoV-2 cross-infections after thyroid surgery occurred in less than 2 in 100 patients, with severe pulmonary manifestations in about 0.5% cases. Although this latter result could be expected (as thyroid surgery is usually not a procedure exposed to the aerodigestive tract, nor a procedure demanding intensive care or long hospital stay) this is a new result since it regards the SARS-CoV-2 infection and concern exist among the scientific community about the feasibility of thyroid surgery during the COVID-19 pandemic [2].

Our study has some limitations. First, seven out of nine studies had a retrospective design resulting in potential selection bias. Second, some factors such as local community SARS-CoV-2 prevalence, different screening methods for SARS-CoV-2 infection, and follow-up length after surgery may have had an impact on the low prevalence of cross-infections. Third, heterogeneity in the size of operated cohorts may have driven some of our results.

A major strength of our study is that this is the first study performing a systematic review on thyroid surgery in the COVID-19 era. Compared to current guidelines and narrative reviews [8–14] we aimed to derive evidence-based results which could encourage the adoption of thyroid surgery despite the ongoing risk of SARS-CoV-2 infection. It has been recently demonstrated that emotional reaction after diagnosis with PTC or an indeterminate thyroid nodule can persist even after receiving education about the excellent prognosis [34]. This will be crucial during the Covid-19 era, when candidates for thyroid surgery desire to undergo this operation despite the risk of transmission of SARS-CoV-2 [35].

## Conclusion

Our systematic review collected the experiences of thyroid surgery teams across several countries, allowing reassuring evidence-based results relative to the feasibility of thyroid surgery in the COVID-19 era. The risk of SARS-CoV-2 transmission after thyroid surgery is relatively low compared to the actual estimates for the general population of most countries of the world [36]. Even considering the global vaccine action plan and the no end in sight to COVID-19 pandemic, our study could serve as a forerunner for regaining control of the growing backlog of planned thyroid surgery due to COVID-19 [37]. Future studies are warranted to obtain more solid data regarding the risk of complications after thyroid surgery during the COVID-19 era. As always, regional and hospital supply status and the safety and personal willingness of our patients should dictate the ultimate timing of thyroid surgery.

## Supplementary Information

Below is the link to the electronic supplementary material.

Supplementary file1 (DOCX 16 kb)

## Abbreviations

PTC: Papillary thyroid cancer
PRISMA: Preferred reporting items for systematic reviews and meta-analyses
CT: Computed tomography

## References

[1] Hojaij FC, Chinelatto LA, Boog GHP (2020). Head and neck practice in the COVID-19 pandemics today: a rapid systematic review. Int Arch Otorhinolaryngol.

[2] Zhao Y, Xu X (2020). Thyroid surgery during COVID-19 pandemic: is it feasible?. Br J Surg.

[3] Nickel B, Glover A, Miller JA (2021). Delays to Low-risk Thyroid cancer treatment during COVID-19-refocusing from what has been lost to what may be learned and gained. JAMA Otolaryngol Head Neck Surg.

[4] Scappaticcio L, Pitoia F, Esposito K (2020). Impact of COVID-19 on the thyroid gland: an update. Rev Endocr Metab Disord.

[5] Tsang VHM, Gild M, Glover A (2020). Thyroid cancer in the age of COVID-19. Endocr Relat Cancer.

[6] Smulever A, Abelleira E, Bueno F (2020). Thyroid cancer in the Era of COVID-19. Endocrine.

[7] Scappaticcio L, Bellastella G, Maiorino MI (2021). Medical treatment of thyrotoxicosis. Q J Nucl Med Mol Imaging.

[8] Shaha AR (2020). Thyroid surgery during COVID-19 pandemic: Principles and philosophies. Head Neck.

[9] Mehanna H, Hardman JC, Shenson JA (2020). Recommendations for head and neck surgical oncology practice in a setting of acute severe resource constraint during the COVID-19 pandemic: an international consensus. Lancet Oncol.

[10] Baud G, Brunaud L, Lifante JC, AFCE COVID Study Group (2020). Endocrine surgery during and after the COVID-19 epidemic: Expert guidelines from AFCE. J Visc Surg.

[11] Jozaghi Y, Zafereo ME, Perrier ND (2020). Endocrine surgery in the Coronavirus disease 2019 pandemic: Surgical Triage Guidelines. Head Neck.

[12] Topf MC, Shenson JA, Holsinger FC (2020). Framework for prioritizing head and neck surgery during the COVID-19 pandemic. Head Neck.

[13] Vrachimis A, Iakovou I, Giannoula E (2020). Endocrinology in the time of COVID-19: management of thyroid nodules and cancer. Eur J Endocrinol.

[14] Boelaert K, Visser WE, Taylor PN (2020). Endocrinology in the time of COVID-19: management of hyperthyroidism and hypothyroidism. Eur J Endocrinol.

[15] Moher D, Liberati A, Tetzlaff J (2009). Preferred reporting items for systematic reviews and meta-analyses: the PRISMA statement. BMJ.

[16] Van Le Q, Ngo DQ, Tran TD (2020). The impact of COVID-19 pandemic on thyroid surgery in Vietnam. Eur J Surg Oncol.

[17] Medas F, Ansaldo GL, Avenia N (2021). Impact of the COVID-19 pandemic on surgery for thyroid cancer in Italy: nationwide retrospective study. Br J Surg.

[18] Glasbey JC, Nepogodiev D, Simoes JFF, COVIDSurg Collaborative (2021). Elective cancer surgery in COVID-19-free surgical pathways during the SARS-CoV-2 pandemic: an international, multicenter, comparative cohort study. J Clin Oncol.

[19] Tagliabue M, Russell B, Moss C (2021). Outcomes of head and neck cancer management from two cancer centres in Southern and Northern Europe during the first wave of COVID-19. Tumori.

[20] Kalita S, Gogoi B, Khaund G (2021). Optimizing airway surgery in COVID 19 Era. Indian J Otolaryngol Head Neck Surg.

[21] Sawka AM, Ghai S, Ihekire O, On behalf of the Canadian thyroid cancer (2021). decision-making in surgery or active surveillance for low risk papillary thyroid cancer during the COVID-19 pandemic. Cancers (Basel).

[22] Bakkar S, Al-Omar K, Aljarrah Q (2020). Impact of COVID-19 on thyroid cancer surgery and adjunct therapy. Updates Surg.

[23] Cai YC, Wang W, Li C (2020). Treating head and neck tumors during the SARS-CoV-2 epidemic, 2019 to 2020: Sichuan Cancer Hospital. Head Neck.

[24] COVIDSurg Collaborative (2020). Head and neck cancer surgery during the COVID-19 pandemic: An international, multicenter, observational cohort study. Cancer.

[25] Lombardi CP, D'Amore A, Grani G (2020). Endocrine surgery during COVID-19 pandemic: do we need an update of indications in Italy?. Endocrine.

[26] Wai KC, Xu MJ, Lee RH (2021). Head and neck surgery during the coronavirus-19 pandemic: The University of California San Francisco experience. Head Neck.

[27] Zhang D, Fu Y, Zhou L (2020). Thyroid surgery during coronavirus-19 pandemic phases I, II and III: lessons learned in China, South Korea, Iran and Italy. J Endocrinol Invest.

[28] Zhao Y, Jin C, Song Q (2021). Surgical management and outcome of patients with thyroid disease during the COVID-19 pandemic. Br J Surg.

[29] Ferrari M, Paderno A, Giannini L (2021). COVID-19 screening protocols for preoperative assessment of head and neck cancer patients candidate for elective surgery in the midst of the pandemic: a narrative review with comparison between two Italian institutions. Oral Oncol.

[30] Medas F, Ansaldo GL, Avenia N, SIUEC Collaborative Group (2021). The THYCOVIT (Thyroid Surgery during COVID-19 pandemic in Italy) study: results from a nationwide, multicentric, case-controlled study. Updates Surg.

[31] Ghai S (2020). Will the guidelines and recommendations for surgery during COVID-19 pandemic still be valid if it becomes endemic?. Int J Surg.

[32] Gao M, Ge M, Ji Q, Cheng R (2017). Chinese Association Of Thyroid Oncology Cato Chinese Anti-Cancer Association. 2016 Chinese expert consensus and guidelines for the diagnosis and treatment of papillary thyroid microcarcinoma. Cancer Biol Med.

[33] Rosato L, Avenia N, Bernante P (2004). Complications of thyroid surgery: analysis of a multicentric study on 14,934 patients operated on in Italy over 5 years. World J Surg.

[34] Pitt SC, Saucke MC, Wendt EM (2020). Patients' reaction to diagnosis with thyroid cancer or an indeterminate thyroid nodule. Thyroid.

[35] Trimboli P, Piccardo A, Cossa A (2021). Patients diagnosed with low-risk thyroid cancer during COVID-19 pandemic: what did they ask surgeons?. Minerva Endocrinol.

[36] World Health Organization. Coronavirus disease (COVID-19) outbreak. https://www.who.int. Accessed 25 Apr 2021.

[37] Carr A, Smith JA, Camaradou J (2021). Growing backlog of planned surgery due to covid-19. BMJ.

