# Supplementary material for: Thyroid surgery during the COVID-19 pandemic: results from a systematic review
Source: J Endocrinol Invest. 2021 Jul 19;45(1):181–8. doi: 10.1007/s40618-021-01641-1 (PMC8288414; doi:10.1007/s40618-021-01641-1)
Supplement: Supplementary file 1 — Supplementary file1 (DOCX 16 kb) [file 40618_2021_1641_MOESM1_ESM.docx]

|  | Ref.  22 | Ref.  23 | Ref.  24 | Ref.  25 | Ref.  26 | Ref.  27 | Ref.  28 | Ref.  29 | Ref.  30 |
| --- | --- | --- | --- | --- | --- | --- | --- | --- | --- |
| 1. Research question. | L | L | L | L | L | L | L | L | L |
| 2. Study population. | L | L | L | L | L | L | L | L | L |
| 3. Participation rate. | NR | NR | NR | NR | NR | NR | NR | NR | NR |
| 4. Groups recruiting. | L | L | L | L | L | L | L | L | L |
| 5. Sample size. | H | H | H | H | H | H | H | H | H |
| 6. Exposure assessment | L | L | L | L | L | L | L | L | L |
| 7. Sufficient timeframe to see an effect. | L | L | L | L | L | L | L | L | L |
| 8. Different levels of the exposure of interest. | L | L | L | L | L | L | L | L | L |
| 9. Exposure measures. | L | L | L | L | L | L | L | L | L |
| 10. **Repeated exposure assessment.** | L | L | L | L | L | L | L | L | L |
| 11. Outcome measures. | H | L | L | L | L | H | H | L | H |
| 12. **Blinding of outcome assessors.** | L | L | L | L | L | L | L | L | L |
| 13. **Follow-up rate.** | L | L | L | L | L | L | L | L | L |
| 14. Statistical analyses. | L | L | L | L | L | L | L | L | L |

**Supplemental Table 1** Risk of bias summary: review authors' judgements on the quality of the 9 included studies.

Ref,reference; L, low risk; H, high risk; NR, not reported
